# Neohesperidin promotes the osteogenic differentiation of bone mesenchymal stem cells by activating the Wnt/-catenin signaling pathway

**DOI:** 10.1186/s13018-021-02468-5

**Published:** 2021-05-21

**Authors:** Yue-wen Chang, Wen-jun Zhu, Wei Gu, Jun Sun, Zhi-qiang Li, Xiao-en Wei

**Affiliations:** Department of Orthopedics, Shuguang Hospital Affiliated to Shanghai University of Traditional Chinese Medicine, No. 185, Puan Road, Huangpu District, Shanghai, 200021 China

**Keywords:** Neohesperidin, Bone mesenchymal stem cells, Wnt/-catenin pathway

## Abstract

**Background:**

Osteoporosis is a common disease in aging populations. However, osteoporosis treatment is still challenging. Here, we aimed to investigate the role of neohesperidin (NEO) in osteoporosis progression and the potential mechanism.

**Methods:**

Bone mesenchymal stem cells (BMSCs) were isolated and treated with different concentrations of NEO (0, 10, 30, 100 M). Cell proliferation was analyzed by cell count kit-8 (CCK-8) assay. RNA-sequencing was performed on the isolated BMSCs with control and NEO treatment. Differentially expressed genes were obtained by R software. Alkaline phosphatase (ALP) staining and Alizarin red staining (ARS) were performed to assess the osteogenic capacity of the NEO. qRT-PCR was used to detect the expression of osteoblast markers.

Western blot was used to evaluate the protein levels in BMSCs.

**Results:**

NEO treatment significantly improved hBMSC proliferation at different time points, particularly when cells were incubated with 30 M NEO (*P* < 0.05). NEO dose-dependently increased the ALP activity and calcium deposition than the control group (*P* < 0.05). A total of 855 differentially expressed genes were identified according to the significance criteria of log_2_ (fold change) > 1 and adj *P* < 0.05. DKK1 partially reversed the promotion effects of NEO on osteogenic differentiation of BMSCs. NEO increased levels of the -catenin protein in BMSCs.

**Conclusion:**

NEO plays a positive role in promoting osteogenic differentiation of BMSCs, which was related with activation of Wnt/-catenin pathway.

## Background

Osteoporosis is common in women, which is characterized mainly by degeneration of microarchitecture of the trabeculae [1]. Osteoporosis patients have a greater risk of osteoporosis-related fracture [2]. One of the critical causes of bone loss is the imbalance between osteoclast (OC)-related bone resorption and osteoblast-related bone reformation [3]. Estrogen replacement treatment (ERT) is considered an effective therapy for menopausal osteoporosis, but it increases the risk of endometrial cancer, breast cancer, and asthma [4]. Bisphosphonates are the current mainstay for the treatment of postmenopausal osteoporosis [5]. However, bisphosphonates exhibit nephrotoxicity, hepatic toxicity, and alimentary canal toxicity.

As such, new safe and affordable drugs are required for the continued treatment and control of osteoporosis [4]. Neohesperidin (NEO) is citrus aurantium extract and belongs to the flavonoids [6]. Flavonoids are well known to exhibit several biological and pharmacological activities including has anti-inflammatory, anti-tumor, and antiviral effects [7, 8]. However, the role of NEO on osteogenic differentiation of BMSCs was unknown. RNA-sequencing technology is a high-throughput sequencing technology which has developed rapidly in recent years [9]. RNA-sequencing technology can be used to detect the differentially expressed genes between different treatment groups [10]. The Wnt/-catenin signaling pathway serves a vital role in regulating osteoblastic differentiation of BMSCs [11, 12]. Moreover, Wnt/-catenin pathway antagonists can be used for the treatment of osteoporosis [13, 14]. However, whether NEO activates the Wnt/-catenin pathway was unknown.

In our study, we found for the first time that NEO promoted osteogenesis by promoting the Wnt/-catenin signaling pathway. The expression of osteogenic-related genes and proteins was measured to evaluate the influence of this pharmacological intervention of NEO on osteogenic differentiation. Our work indicates that NEO could be a promising therapeutic option for osteolytic bone diseases. Additionally, the therapeutic effect of NEO on preventing bone loss in vivo was confirmed by animal experiments. Data from estrogen deficiency-induced osteoporosis mouse models indicated that the NEO might be a potential curing option for osteoporosis. Although several biological effects of NEO have been reported, its effects on promoting osteogenesis have yet to be discovered.

Therefore, in the present study, we aimed to investigate the effects of NEO on promoting osteogenic differentiation and exact mechanisms in human bone mesenchymal stem cells.

## Methods and materials

### Cell culture

BMSCs were harvested from the human bone marrow as previously described [15]. BMSCs were cultured into alpha-modified minimal essential medium (-MEM) containing 10% FBS and 1% antibiotics. Then, -MEM which was changed per 2 days. Osteogenic induction was performed by replacing the medium with an osteogenic medium (containing 10-nM dexamethasone, 10-mM -glycerophosphate, and 50g/mL vitamin C) (Sigma, USA).

### The efficacy and toxicity evaluation of NEO

BMSCs were seeded in plates (96-well plate) at a concentration of 510^3^ cells per well and cultured in -MEM medium (the component of -MEM medium has mentioned above). Meantime, NEO was added to the cultured cells at varying concentrations (0, 10, 30, and 100 M). The medium was changed per 2 days. BMSCs were treated with varying concertation of NEO (0, 10, 30, and 100 M) for 1, 3, 5, and 7 days, and then, a 10-l CCK-8 solution (Solarbio, Beijing, China) was added into wells, then cells were incubated for another 4 h. The absorbance at 450 nm was measured by the enzyme-linked immunosorbent assay (Thermo Fisher Scientific, Waltham, MA, USA).

### ALP and ARS

ALP staining was monitored using an ALP histochemical diagnostic kit (Beyotime, China) according to the manufacturers protocol. In brief, BMSCs were fixed by 4% paraformaldehyde after 7days of osteogenic induction. After three washes with phosphate-buffered saline (PBS), staining was performed using NBT/BCIP. Then, an equal volume of distilled water was added to abort the reaction. ARS was performed as previously described [16]. BMSCs were fixed by 4% paraformaldehyde after 14days of osteogenic induction. After three washes with PBS, staining was performed using 0.1% ARS solution (Solarbio, Beijing, China). The results were photographed under a Zeiss microscope (Zeiss, Oberkochen, Germany).

### RNA sequencing

BMSCs were cultured and treated with control or NEO (30 M) as described above. RNA-sequencing analysis was performed by the Shanghai Biotechnology Corporation (Shanghai, China). In brief, the total RNA was extracted using an RNeasy Mini kit (QIAGEN, Hilden, Germany) and was reverse transcribed into complementary DNA (cDNA). Paired-end libraries were synthesized by using a TruSeq RNA Sample Preparation Kit (Illumina, USA) according to the TruSeq RNA Sample Preparation Guide.

The cleaved RNA fragments are copied into the first-strand cDNA using reverse transcriptase and random primers. Differential expression gene (DEG) analysis for mRNA was performed using R package edge R [17]. DEGs with log_2_(fold change) value >1 and adj *p* value <0.05, considered as significantly modulated, were retained for further analysis. A volcano plot and heatmap for the differentially expressed genes were generated with ggplot2 and heatmap in R respectively [18]. Next, the enriched Gene Ontology (GO) terms and Kyoto Encyclopedia of Genes and Genomes (KEGG) pathways were performed by using the clusterProfiler R package [19]. To predict protein-protein interactions, Search Tool for the Retrieval of Interacting Genes (STRING) database was used (https://string-db.org) [20]. Also, Molecular Complex Detection (MCODE), one plug-in unit of Cytoscape, was set to screen the models of PPI network.

### Quantitative RT-PCR

TRIzol reagent (Invitrogen) was used to extract total RNA from the cells, and the RNA concentration and purity were determined by ND-1000 spectrophotometer. Revert Aid First-Strand cDNA Synthesis Kit (Fermentas, USA) was used to reverse sample RNA from total RNA into cDNA and RT-PCR. Primer list as follow: GAPDH, forward: 3-GGAGAAAGCTGCTAA-5, Reverse: 3-ACGACCTGGTCCTCGGTGTA-5; -catenin, Forward: 3-GTGTGGAGTGCCTGATGTG-153 5, Reverse: 3-CTGCTTGACCCTTGGAGAC-5; TCF7, Forward: 3TTCCAACCCTGCTACTGC-5, Reverse: 3-TGCCTGTCACCTCCAAG-5; Lef1, Forward: 3-CTTCTGGCTCACGCTTTTC-5, Reverse: 3-TTGGGGTCTTCATCTCCTG-5. c-myc, Forward: 3-GAAGTGGCTCACGCTTTTC-, Reverse: 3-AAGVGGTCTACTACGCCAG-5, RUNX2, 5-AAGGAAGCTTGGCGTTGTGA-3; reverse 5-GAGAGGTGAGGAGTCTTATG-3, BMP2, Forward: 5-AACACGCTGCCTGTCTACACT-3; Reverse: 5-CAGTGCAGGGTCCGAGGT-3 and Osteocalcin, Forward: 5-GACCCCTTTACTCTGACCCC-3, Reverse: 5-AGGCTCCAGTGAATTCGGAA-3. The relative expression of each gene was detected and calculated.

### Western Blot analysis

The intracellular protein was collected by digestion and centrifugation, washed twice with PBS, and the cell pellet was collected by centrifugation. A certain amount of lysate was added and lysed on ice for 30 min. After lysing, the total protein was collected by centrifugation at 12000 r/min for 15 min at 131 4C. A certain amount of protein loading buffer was added and the mixture was denatured at 100C for 15 min, and then, polyacrylamide gel electrophoresis (12%, SDS-PAGE) was performed. After electrophoresis, the proteins were transferred to a 4.5-m polyvinylidene fluoride (PVDF) membrane. The PVDF membrane was blocked with 5% skimmed milk powder for 1 h at room temperature, and a primary antibody -catenin (Abcam, 1:1000), p--cateinin (Abcam, 1:1000), and GAPDH (Abcam, 1:1000) were added to incubate overnight. After incubation, the PVDF membrane was washed 3 times with TBST buffer (TRIS-HCl balanced salt buffer + Tween) and then incubated with the secondary antibody (IgG H&L (HRP), Abcam, 1:2000) at room temperature in the dark for 1 h. The bands were then washed three times with TBST buffer, the membrane was scanned with an ODYSSEY infrared imaging system, and the molecular weight and optical density values of the target bands were analyzed using a gel image processing system.

### Statistical analysis

In the results section, all of the quantitative data are presented as the mean standard deviation. Statistical analyses were conducted using one-way analysis of variance (ANOVA) followed by Tukeys post hoc test with GraphPad Prism 8. All data are demonstrated as the means SDs; *P< 0.05 comparing to the control.

## Results

### NEO increased hBMSC proliferation and osteogenic differentiation

**Fig. 1:**
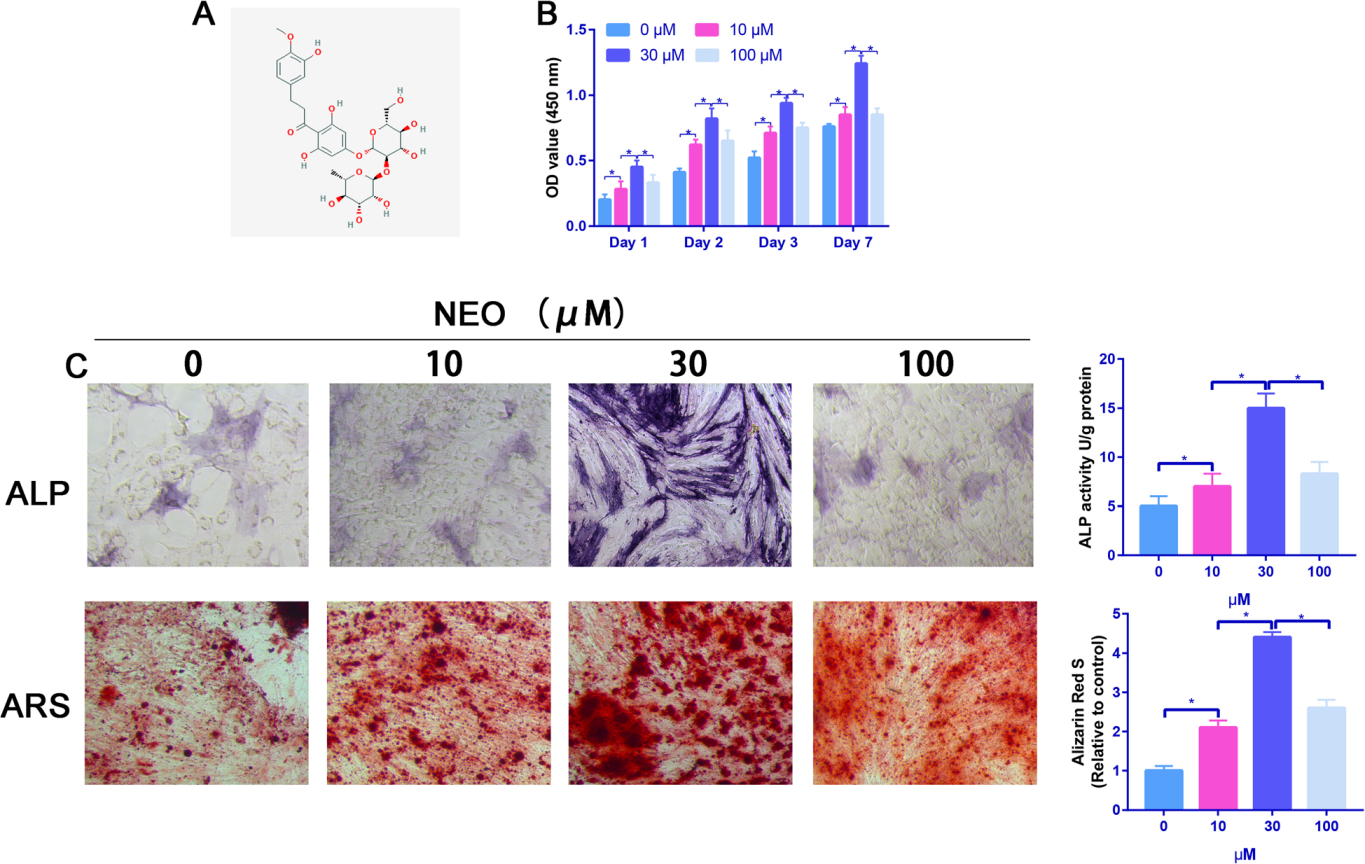
NEO promoted the proliferation and osteogenic differentiation of BMSCs. **a** A 2D structure of NEO. **b** CCK-8 assay to assess the proliferation ability of NEO for BMSCs. **c** ALP and ARS staining of control and different concentration of NEO

BMSCs were incubated in an osteogenic induction medium with or without NEO at different concentrations (0, 10, 30, and 100 M) for 1, 2, 3, and 7 days. According to the results of the CCK-8 assay, the NEO treatment significantly improved hBMSC proliferation at different time points, particularly when cells were incubated with 30-M NEO (*P* < 0.05) (Fig. 1b). Then, ALP and Alizarin red staining were used to determine the effect of NEO on the osteogenic differentiation of hBMSCs. Results showed that NEO dose-dependently increased the ALP activity and calcium deposition than the control group (Fig. 1c).

**Fig. 2:**
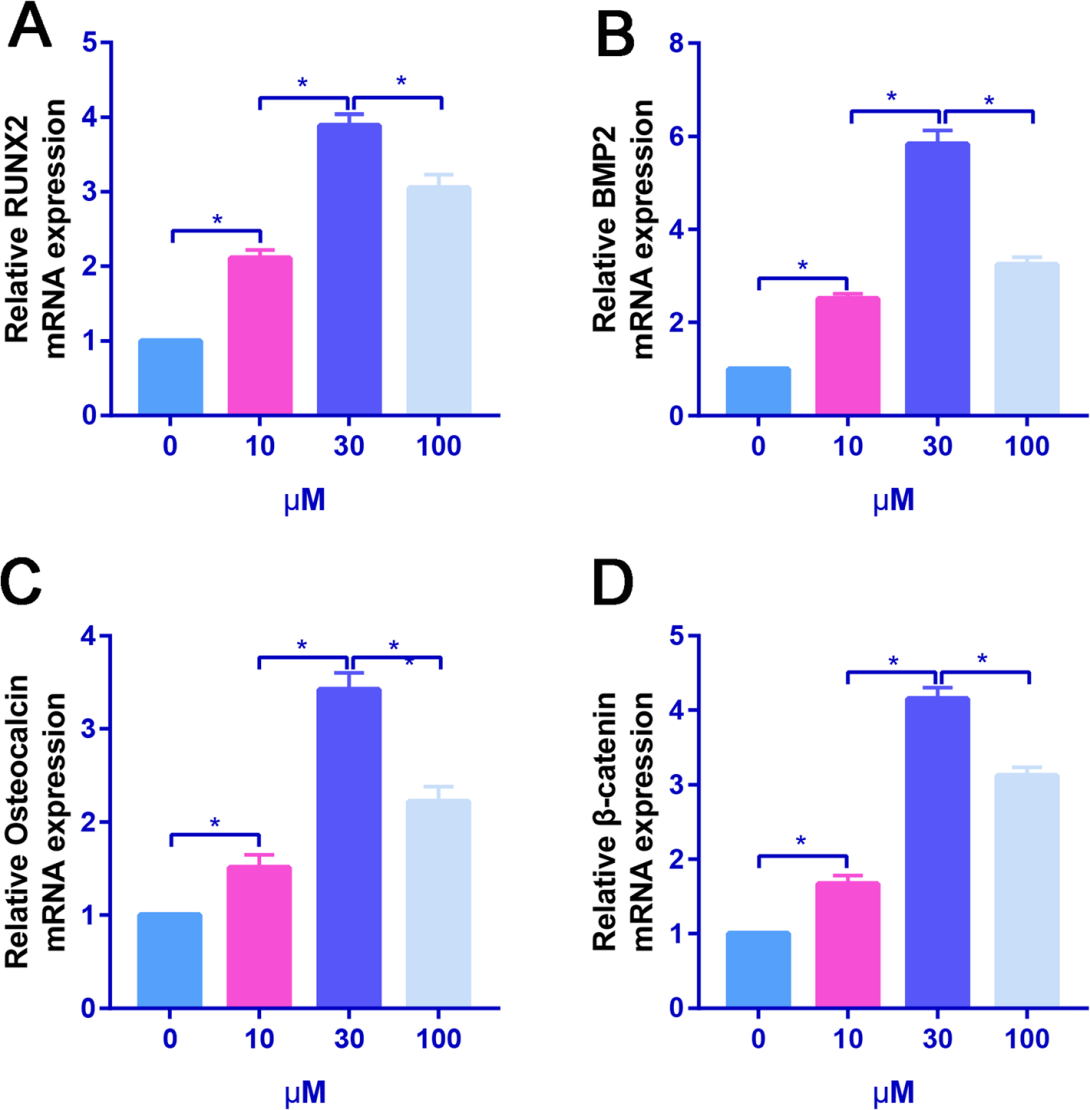
A NEO dose-dependently increased the osteogenesis-related genes. **a** Relative expression of RUNX2 expression in control and NEO treatment groups. **b** Relative expression of BMP2 expression in control and NEO treatment groups. **c** Relative expression of osteocalcin expression in control and NEO treatment groups. **d** Relative expression of -catenin expression in control and NEO treatment groups

The expression levels of osteogenesis-related genes (Runx2, Osteocalcin, and BMP2) were assessed by real-time PCR. NEO dose-dependently increased the osteogenesis-related genes (Runx2, Osteocalcin, and BMP2) of hBMSCs than that of the control group (Fig. 2ac). Moreover, NEO significantly increased the -catenin expression, which suggested that NEO could activate the Wnt--catenin signaling pathway.

### Differentially expressed genes between NEO and control

**Fig. 3:**
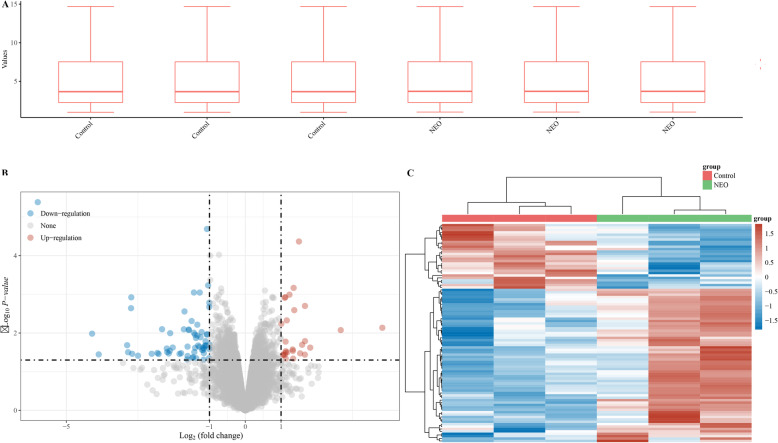
**a** The data were normally distributed *after normalization*. **b** Volcano plot of the differentially expressed genes between NEO and control groups. **c** Heatmap pf the differentially expressed genes between NEO and control groups

The raw data was normalized and the final expression value in each group was in a identical level (Fig. 3a). A total of 83 differentially expressed genes were identified according to the significance criteria of log_2_ (fold change) > 1 and adj *P* < 0.05.

Among these, 58 mRNAs were upregulated and 25 were downregulated. The volcano plot and heatmap of the differentially expressed mRNAs can be seen in Fig. 3b, c**,** respectively.

**Fig. 4:**
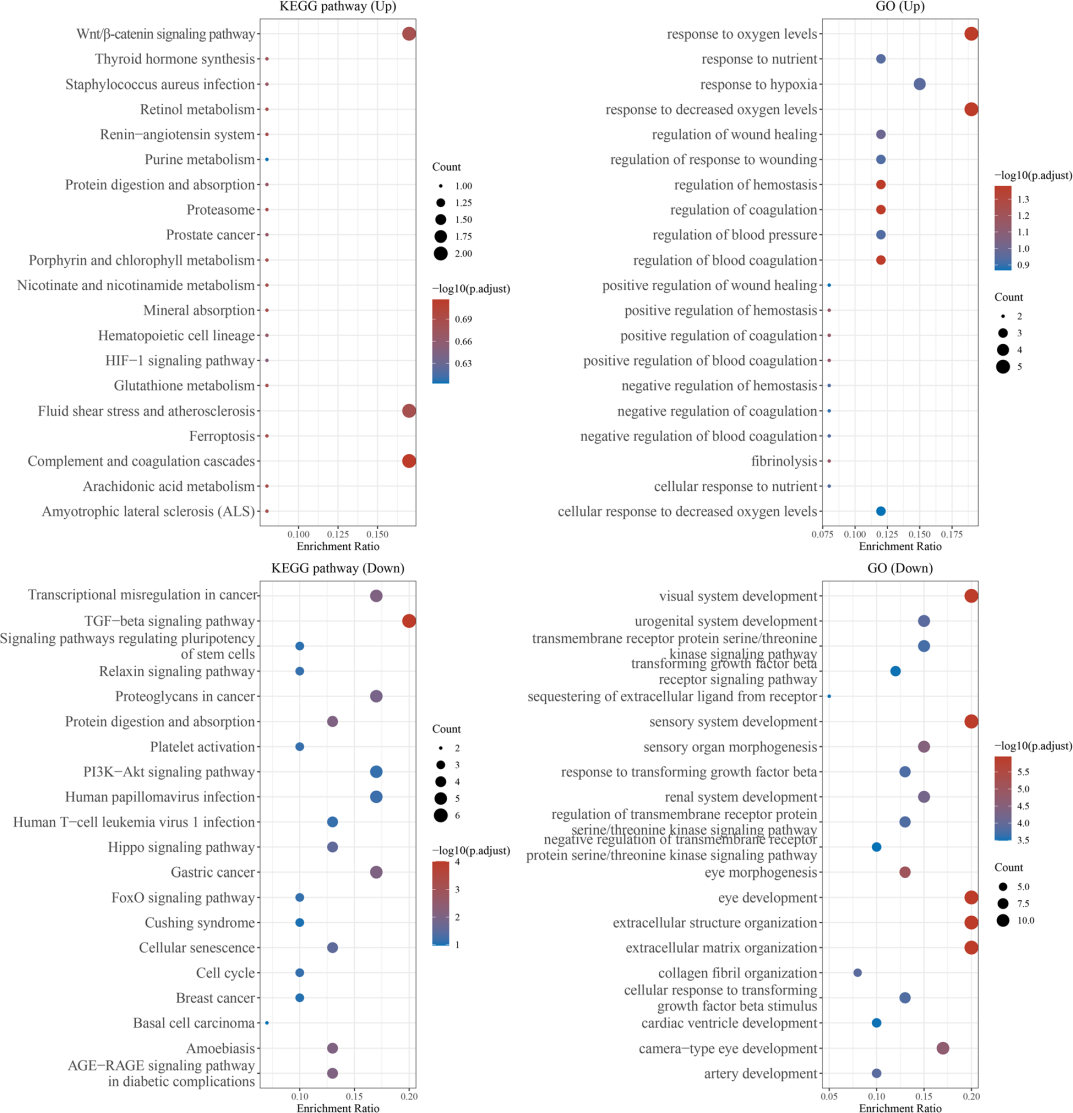
Gene Ontology (GO) and Kyoto Encyclopedia of Genes and Genomes (KEGG) of the differentially expressed genes between control and NEO treatment groups, *P<0.05.

Upregulated expressed genes were mainly enriched into Wnt/-catenin signaling pathway, Thyroid hormone synthesis, Staphylococcus aureus infection, Retinol metabolism, and the Renin-angiotensin system (Fig. 4). Downregulated expressed genes were mainly enriched into transcriptional misregulation in cancer, TGF-beta signaling pathway, signaling pathway regulation pluripotency of stem cells, Relaxin signaling pathway, Protein digestion, and absorption and platelet activation (Fig. 4).

Upregulated expressed genes were mainly enriched in response to oxygen levels, response to nutrient, response to hypoxia, response to decreased oxygen levels, and regulation of wound healing (Fig. 4).

Downregulated expressed genes were mainly enriched into visual system development, urogenital system development, transmembrane receptor protein serine/threonine kinase signaling pathway, transforming growth factor-beta receptor signaling pathway, and sequestering of the extracellular ligand from the receptor (Fig. 4).

### DKK1 partially reversed the promotion effects of NEO on osteogenic differentiation of BMSCs

**Fig. 5:**
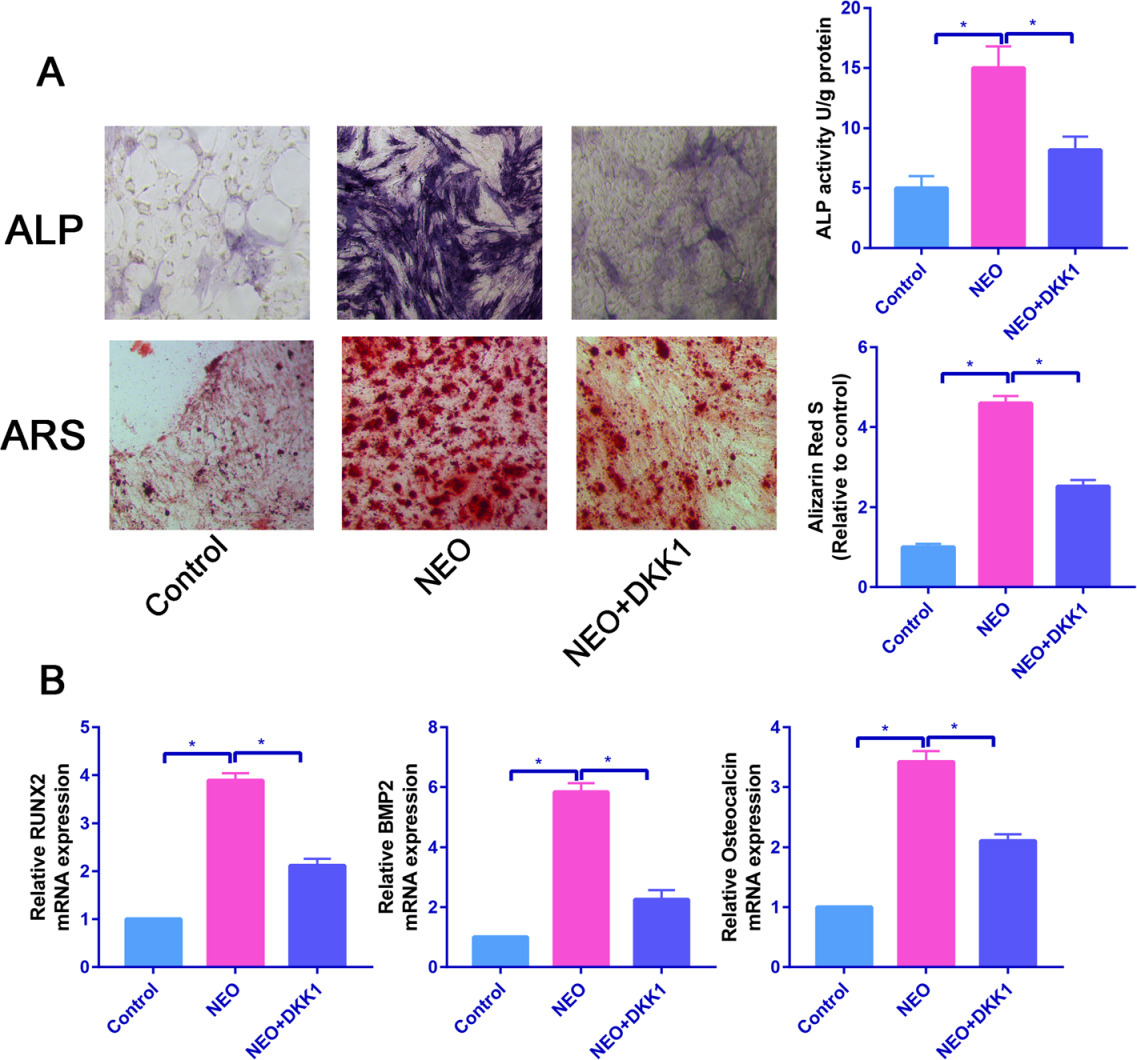
DKK1 partially reversed the promotion effects of NEO on osteogenic differentiation of BMSCs. **a** ALP and ARS staining of control, NEO, and NEO+DKK1 groups. **b** Relative osteogenesis-related genes (Runx2, osteocalcin, and BMP2) in the control, NEO, and NEO+DKK1 groups, *P<0.05

Consistent with previous results, NEO significantly increased the ALP activity and calcium deposition, while these effects of NEO were partially blocked by Wnt--catenin signaling pathway inhibitor, DKK1 (Fig. 5a).

Then, we assessed the osteogenesis-related genes (Runx2, osteocalcin, and BMP2) in control, NEO, and NEO+DKK1 groups. We found that NEO significantly increased osteogenesis-related genes (Runx2, osteocalcin, and BMP2) expression, while these upregulated expressions were partially reversed by DKK1 (Fig. 5b).

### NEO increased levels of the -catenin protein in BMSCs

**Fig. 6:**
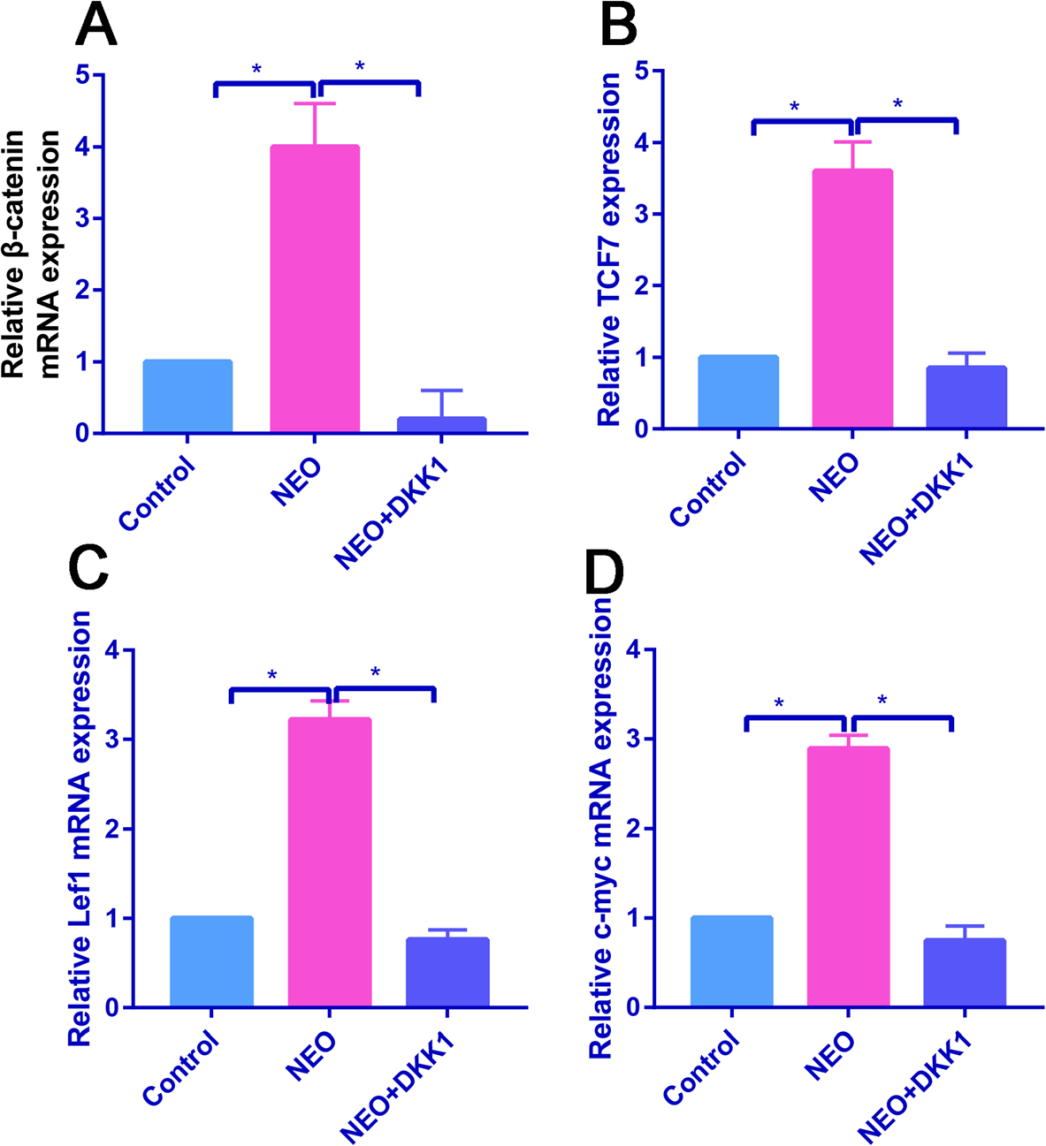
DKK1 partially reversed the promotion effects of NEO on -catenin expression. **a** Relative expression of -catenin expression in the control, NEO, and NEO+DKK1 groups. **b** Relative expression of TCF7 expression in the control, NEO, and NEO+DKK1 groups. **c** Relative expression of Lef1 expression in the control, NEO, and NEO+DKK1 groups. **d** Relative expression of c-myc expression in the control, NEO, and NEO+DKK1 groups, *P<0.05

qRT-PCR was performed to detect -catenin, TCF7, Lef1, and c-myc mRNA levels in control, NEO, and NEO+DKK1 groups. Results found that NEO significantly increased -catenin, TCF7, Lef1, and c-myc expression than the control group (Fig. 6ad, *P*<0.05), while these effects could be partially reversed by DKK1 (Fig. 6ad, *P*<0.05).

**Fig. 7:**
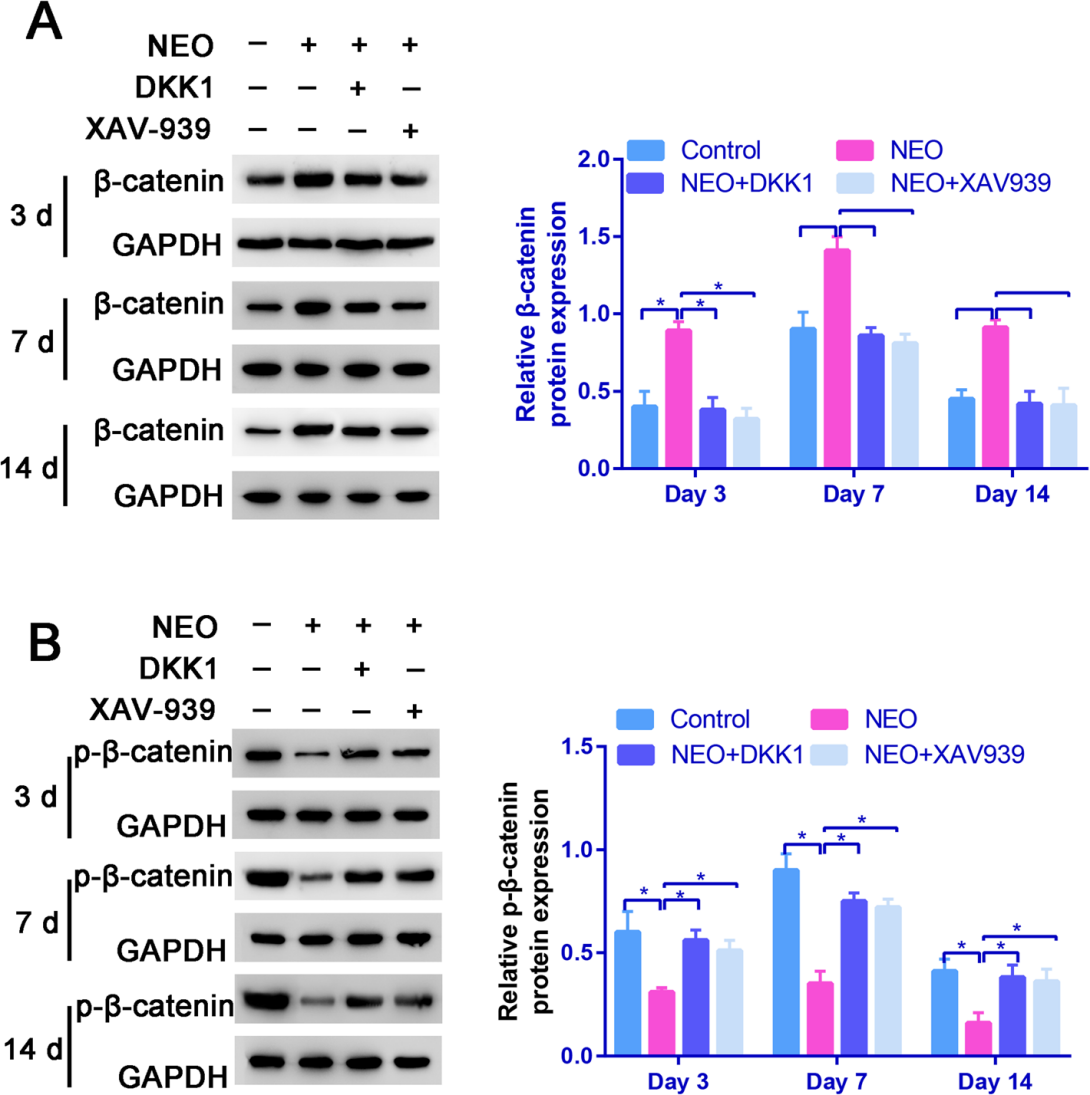
NEO increased levels of the -catenin protein in BMSCs. **a** Relative -catenin expression in the control, NEO, and NEO+DKK1 groups. **b** Relative p--catenin expression in the control, NEO, and NEO+DKK1 groups, *P<0.05

We then conducted Western Blotting to examine the levels of the -catenin and p--catenin proteins and investigate whether NEO increased hBMSC osteogenesis via the Wnt/-catenin pathway. NEO increased -catenin levels on days 3, 7, and 14 compared to the control group (Fig. 7a).

Moreover, p--catenin levels showed the opposite trend to -catenin levels, as expected (Fig. 7b).

## Discussion

In this study, we firstly revealed that NEO could promote osteogenic differentiation of BMSCs through Wnt/-catenin signaling pathway. Moreover, we performed a differentially expressed gene between NEO and control groups from the RNA-sequencing data. NEO may be an effective addition to osteoporotic therapy.

CCK-8 assay was carried out to determine the optimum concentration of NEO. The most suitable final concentration of NEO was found to be 30 M. To demonstrate the function of NEO in osteogenic differentiation, we performed ALP and ARS staining assays. Results found that NEO significantly increased the ALP activity and calcium deposition. It is known that ALP activity and calcium deposition are osteogenic markers indicating cell differentiation ability [21].

NEO, which is a natural flavanone glycoside with numerous pharmacological properties, including antiviral, antioxidant, anti-inflammation, and antitumor [22, 23]. However, the role of NEO on osteogenic differentiation of BMSCs was unknown. BMSCs are a kind of adult stem cells derived from mesoderm with strong proliferation, expansion ability, and multidirectional differentiation potential [24, 25]. They exist in the bone marrow and have the adherent ability and can differentiate into osteoblasts, adipocytes, and other cells [26, 27] through the menopausal transition in women exhibited by an increase in fat accumulation as well as impaired osteogenic differentiation potentials [28]. Therefore, the promotion of osteogenic differentiation can help inhibit the development of osteoporosis.

In order to explore the mechanisms of NEO during the osteogenic differentiation of BMSCs, we performed RNA sequencing analysis to determine differentially expressed genes affected by NEO. A total of 855 differentially expressed genes were identified according to the significance criteria of log_2_ (fold change) > 1 and adj *P* < 0.05. KEGG pathway enrichment analysis revealed that these differentially expressed genes mainly enriched in Wnt--catenin signaling pathway.

Several signaling pathways have been identified that participated in osteogenic differentiation of BMSCs, such as PI3K/Akt signaling and Wnt/beta-catenin pathway [29,33]. Loss of function mutations in the Wnt co-receptor LRP5 results in severe osteoporosis [34].

In this study, NEO activated -catenin and induce osteogenic differentiation of BMSCs, which in turn promote the expression of osteoblast markers. This NEO-induced osteogenic activity was obviously blocked by DKK1 or XAV939. Wnt/beta-catenin pathway not only stimulates the osteogenic differentiation of BMSCs, but also induces the maturation of BMSCs into osteoblasts [35]. Previous studies suggested that inactivation of Wnt/beta-catenin will switch the osteogenesis of MSCs to chondrogenesis [36]. Taken together, we firstly identified that NEO stimulated osteogenic differentiation of BMSCs through Wnt/beta-catenin pathway. NEO has no toxicities or adverse effects at the animal level [37].

Several important limitations in this study are worth mentioning. First, we did not perform in vivo animal study to identify the role of NEO to prevent the osteoporosis progression. Second, the optimal dose of NEO needed for more studies to identify. Last, the receptor of NEO needed for more studies to identify.

## Conclusion

In conclusion, NEO enhanced the proliferation and osteogenic differentiation of BMSCs. NEO promoted the osteogenesis of BMSCs by activating the Wnt signaling pathway. The current study describes a promising therapeutic agent for patients with osteoporosis.

## Data Availability

All the data pertaining to the present study are willing to share upon reasonable request.

## Abbreviations

NEO: Neohesperidin
BMSCs: Bone mesenchymal stem cells
CCK-8: Cell count kit-8
ALP: Alkaline phosphatase
ARS: Alizarin red staining
OC: Osteoclast
ERT: Estrogen replacement treatment
-MEM: Alpha-modified minimal essential medium
PBS: Phosphate-buffered saline
DEGs: Differential expression genes
GO: Gene Ontology
KEGG: Kyoto Encyclopedia of Genes and Genomes
STRING: Search Tool for the Retrieval of Interacting Genes
MCODE: Molecular Complex Detection
PVDF: Polyvinylidene fluoride
ANOVA: One-way analysis of variance
